# Long-Term Outcomes Following Inguinal Hernia Repair With Mesh Performed by Medical Doctors and Surgeons in Ghana

**DOI:** 10.1097/AS9.0000000000000460

**Published:** 2024-07-15

**Authors:** Jessica H. Beard, Michael Ohene-Yeboah, Emmanuel S. Kasu, Nelson Affram, Stephen Tabiri, Joachim K. A. Amoako, Francis A. Abantanga, Jenny Löfgren

**Affiliations:** * From the Department of Surgery, Division of Trauma Surgery and Surgical Critical Care, Lewis Katz School of Medicine, Temple University, Philadelphia, PA; † Department of Surgery, School of Medicine and Dentistry, University of Ghana, Accra, Ghana; ‡ Department of Public Health, Volta Regional Hospital, Ho, Ghana; § Department of Surgery, Ho Teaching Hospital, Ho, Ghana; ‖ Department of Surgery, School of Medicine and Health Sciences, University for Development Studies, Tamale, Ghana; ¶ Department of Molecular Medicine and Surgery, Karolinska Institutet, Stockholm, Sweden.

**Keywords:** global surgery, hernia repair, inguinal hernia, surgical outcomes, task sharing

## Abstract

**Objective:**

To assess long-term outcomes following inguinal hernia repair with mesh performed by medical doctors and surgeons in Ghana.

**Background:**

Task sharing of surgical care with nonsurgeons can increase access to essential surgery. Long-term safety and outcomes of task sharing are not well-described for hernia repair.

**Methods:**

This prospective cohort study was conducted in Ho, Ghana. After completing a training course, 3 medical doctors and 2 surgeons performed inguinal hernia repairs with mesh on men with primary, reducible hernias. The primary outcome of this study was hernia recurrence at 5 years. The noninferiority limit was 5 percentage points. Secondary endpoints included pain and self-assessed health status at 5 years.

**Results:**

A total of 242 operations in 241 participants were included, including 119 hernia repairs performed by the medical doctors and 123 performed by the surgeons. One hundred and sixty-nine participants (70.1%) were seen in follow-up at 5 years, 29 participants (12.0%) had died and 43 (17.8%) were lost to follow-up. The overall 5-year recurrence rate was 4.7% (n = 8). The absolute difference in recurrence rate between the medical doctor group (2 [2.3%]) and the surgeon group (6 [7.3%]) was −5.0 (1-tailed 95% confidence interval, −10.5; *P* = 0.06), demonstrating noninferiority of the medical doctors. Participants experienced improvements in groin pain and self-assessed health status that persisted at 5 years.

**Conclusions:**

Long-term outcomes of elective mesh inguinal hernia repair in men performed by medical doctors and surgeons in Ghana were excellent. Task sharing is a critical tool to address the substantial morbidity of unmet hernia surgery needs in Ghana.

## INTRODUCTION

Inguinal hernia is the most common general surgical condition globally.^1–4^ Case volumes do not meet the need for surgery in many low- and middle-income countries (LMICs), leading to a significant backlog of patients waiting for essential surgical procedures, including inguinal hernia repair.^1,5–7^ Low surgical output in LMICs is driven by limited material and human resources, including a critical shortage of specialist surgeons.^8,9^ In recent years, access to inguinal hernia repair has increased in some LMICs; however, standard Lichtenstein inguinal hernia repair with mesh is still not widely available in low-resource settings.^7,10^

In sub-Saharan Africa, where the availability of surgeons is most limited, task sharing of surgical procedures with nonsurgeons is widely practiced.^7,11–15^ Previous studies have demonstrated that task sharing is a safe, effective, and cost-effective way to increase access to surgical care in LMICs.^14,16–20^ To utilize task sharing to build capacity for high-quality inguinal hernia repair in Ghana, the Ghana Hernia Society has developed a standardized training course in Lichtenstein mesh repair for medical doctors and surgeons. The safety and effectiveness of task sharing for mesh hernia repair in Ghana were previously assessed in a prospective cohort study conducted by our research team.^19^ Following completion of the Ghana Hernia Society training course, medical doctors and surgeons performed inguinal hernia repair with mesh on men with a primary reducible hernia at a regional hospital in Ghana. Of note, more than half of the hernias repaired in the cohort (52.8%) were inguinoscrotal hernias. Outcomes were similar in both provider groups. The overall 1-year recurrence rate among the study participants was 1.8% with 1 recurrence in the medical doctor group (0.9%) and 3 recurrences in the surgeon group (2.8%).^19^ A similar randomized controlled trial completed in a rural first-level hospital in Sierra Leone demonstrated the safety and effectiveness of task sharing of inguinal hernia repair with mesh with associate clinicians, who are surgical providers with an educational level between that of a nurse and medical doctor.^20^ In that study, the recurrence rate at 1 year was 0.9% among 109 men with primary reducible hernia operated on by associate clinicians.^20^ Together these studies indicate that associate clinicians, medical doctors, and surgeons can be trained to perform elective inguinal hernia repair with mesh in men with similar outcomes in low-resource settings.^19,20^

While there is increasing evidence that surgical task sharing with adequate training and oversight is safe and effective, longer-term outcomes following surgical procedures performed by nonsurgeons have not been closely examined. In addition, studies evaluating outcomes of inguinal hernia repair with mesh with long-term follow-up in sub-Saharan Africa are limited.^21^ The aim of the present study was to evaluate the long-term outcomes following inguinal hernia repair with mesh performed by medical doctors and surgeons in Ghana. In this study, we followed up on patients who participated in the original cohort study in Ghana 5 years postoperatively.^19^ We hypothesized that medical doctors would be noninferior to surgeons in terms of recurrence, chronic pain, and patient satisfaction at 5 years.

## METHODS

### Study Design

This is a prospective cohort study with a noninferiority design comparing long-term outcomes following inguinal hernia repair with mesh performed by medical doctors and surgeons in Ghana. Ethical approval was obtained from the Ghana Health Service Ethical Review Committee and the University of Pennsylvania Institutional Review Board. Participants were enrolled in the study after giving written informed consent, and they were compensated for transportation costs to follow-up visits. The study was conducted from February 2017 to February 2023. A full description of the design and procedures along with 2-week and 1-year outcomes has been previously reported.^19^ The original study design included provision for a 3-year follow-up; however, this was delayed until approximately 5 years postoperatively due to the COVID-19 pandemic.

### Surgical Training Program and Materials

In 2017, The Ghana Hernia Society led a 2-week training course for 3 medical doctors and 2 surgeons in tension-free anterior mesh hernia repair according to the Lichtenstein technique.^19,22^ All medical doctors and surgeons trained were proficient in tissue inguinal hernia repair and 1 surgeon had experience in mesh repair. The lifetime experience in inguinal hernia repair for the medical doctors and surgeons who participated in the course was between 60 and 500 surgeries. The medical doctors had completed medical school followed by a 2-year internship but had no formal training in surgery. The surgeons had completed 6 years of postgraduate training in general surgery. The training course included lectures on hernia epidemiology, anatomy of the groin, hernia diagnosis, Lichtenstein technique and local anesthesia administration, and hands-on technical training with graded responsibility. Two trainers independently evaluated each medical doctor and surgeon’s competence using the American Board of Surgery Operative Performance Assessment Form for open inguinal hernia.^23,24^ Scores of 4 or 5 out of 5 were required to pass the course.

Following completion of the course, the 5 medical doctors and surgeons performed Lichtenstein hernia repairs using local anesthesia and a low-cost commercial polypropylene mesh purchased for $11 in Ghana. Patients were assigned to surgical provider based on operating room schedule and provider availability and the surgeries were completed over 6 months during 4 surgical camps.^25^ Patients were given preoperative oral amoxicillin-clavulanate and the World Health Organization Surgical Safety Checklist was used during each case.^26^ All hernia repairs were performed under local anesthesia using the Lichtenstein infiltration technique except in 1 case where general anesthesia was required because of patient intolerance and an especially large hernia.^27^ The program paid one-half of the medical fees for patients ($30–$60) and provided the mesh.

### Setting, Participants, and Procedures

The study was conducted at Volta Regional Hospital in Ho, Ghana, a 306-bed referral hospital managed by the Ghana Health Service. Ghana is a lower-middle-income country in West Africa and ranks 133 of 190 countries and territories on the United Nations Human Development Index.^28^ All patients who underwent operations by the medical doctors and surgeons trained by the program were invited to participate and all agreed to take part and gave consent. The study population consisted of men aged 18 years and older with primary, reducible inguinal hernia. Patients with bilateral hernias were offered repair on the most symptomatic side. One patient with bilateral hernias had 1 hernia repaired initially and then returned to have his contralateral hernia repaired at a subsequent time, contributing data postoperatively about 2 hernia surgeries. Patients with alcohol or drug abuse, coagulopathy, and/or the American Society of Anesthesiologists (ASA) classification score of 3 and above were excluded from participation. Only men were included as they represent the majority of patients with inguinal hernia, their anatomy is less varied than in women, and the risk of adverse outcomes such as chronic pain is lower compared to women.^29^

Study participants were interviewed in Ewe, Twi, or English based on their preference. Data were collected at 5 time points: before the surgery, immediately after the surgery, and at 2 weeks, 1 year, and 5 years following the surgery. Preoperative data included participant characteristics, medical history, self-assessed health status, hernia pain assessment, and physical examination. Perioperative data included information regarding the surgical procedure. Postoperative data included an interview to assess patient satisfaction and self-identified complications along with self-assessed health status, hernia pain assessment, and physical examination. A health thermometer scale ranging from 0 (the worst imaginable health) to 100 (best imaginable health) was used to determine the participants’ self-perceived health. Pain was assessed using the validated Inguinal Pain Questionnaire (IPQ) for groin symptoms. The IPQ is a 7-level scale that ranges from a score of 1, which represents no pain, to a score of 7, which represents severe pain that requires immediate medical attention.^30^ The health thermometer scale and IPQ were used preoperatively and at all follow-up visits. A medical doctor or surgeon blinded to the identity of the operating provider performed the postoperative physical examinations to assess for hernia recurrence and complications.

Prior to the 5-year follow-up, participants were contacted by phone and given a date and time to come to Volta Regional Hospital for review. Study staff offered several reminders to participants to attend the 5-year follow-up. In cases where participants could not be reached or if they were unable to travel, home visits were undertaken. In case of death of a study participant, his next of kin was contacted and interviewed about the date and circumstances of the death.

### Outcomes

The primary outcome of this study was hernia recurrence within 5 years of the operation. Recurrence was defined as a palpable mass with a cough impulse on the same side as the repair. A hernia was not easily detected on physical examination for one of the study participants who had a recurrent hernia at the 1-year follow-up. For this participant, a groin ultrasound was performed and recurrent hernia was observed. Secondary outcomes recorded at 5 years included patient satisfaction, pain according to the IPQ, and self-assessed health status.

### Statistical Analysis

Data were analyzed using descriptive and comparative statistical methods. Continuous outcomes are presented as mean with standard deviation while nominal outcomes are presented as numbers with percentages. The 2 study arms were compared using independent sample *t* test and 1-way analysis of variance for continuous variables along with Pearson chi-square test or Fischer exact test for counts. Absolute differences with 95% confidence interval were calculated using the following formula:

Confidence interval = (p1 − p2) ± z*√(p1(1 − p1)/n1 + p2(1 − p2)/n2)

where

- p1, p2: sample 1 proportion, sample 2 proportion
- z: the z-critical value based on the confidence level
- n1, n2: sample 1 size, sample 2 size

The initial sample size of 242 was calculated based on a noninferiority design with assumptions of 80% power, 5% significance level, 5% noninferiority limit, recurrence rate of 2%, and 20% loss to follow-up.^19^ A *P* < 0.05 was considered statistically significant. The data were analyzed using IBM SPSS version 27 and R Version 4.2.1.

## RESULTS

**FIGURE 1.**
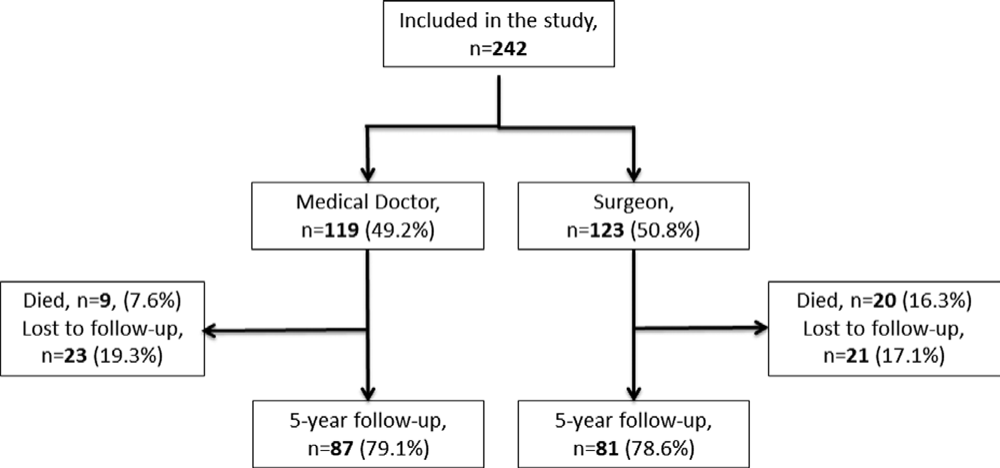
Flowchart of patients included in the study.

A total of 242 inguinal hernias in 241 patients were repaired, including 119 hernia repairs performed by the medical doctors and 123 performed by the surgeons. At the 5-year follow-up, 169 participants (70.1%) completed the interview and physical examination, 29 participants (12.0%) had died, and 43 participants (17.8%) were lost to follow-up (Fig. 1). Baseline characteristics of the study participants who completed the 5-year follow-up are compared to participants who died or were lost to follow-up in Table 1. Participants who died were significantly older and more likely to be smokers than those who completed or were lost to follow-up. Of the participants who completed the 5-year follow-up, there were no significant differences in baseline characteristics, including ASA score, preoperative IPQ, preoperative self-assessed health status, and proportion inguinoscrotal hernias between medical doctor and surgeon groups.

**TABLE 1.** Baseline Characteristics of Participants Who Completed 5-Year Follow-Up Compared to Those Who Died or Were Lost to Follow-Up

Characteristic	Completed Follow-Up, n = 169	Died, n = 29	Lost to Follow-Up, n = 44	*P*
Age at operation, mean (SD), y	51.3 (15.8)	61.3 (11.3)	49.5 (19.5)	0.005
ASA score 1, n (%)	140 (82.8)	20 (69.0)	38 (88.4)	0.174
Body mass index, mean (SD), kg/m^2^	21.9 (2.9)	20.6 (3.3)	21.9 (3.3)	0.112
Inguinoscrotal hernia, n (%)	86 (50.9)	18 (62.1)	24 (54.5)	0.522
Smoker, n (%)	9 (5.3)	5 (17.2)	4 (9.1)	0.013
Self-assessed health status score before operation (0–100), mean (SD)	68.7 (13.4)	66.4 (13.8)	68.1 (15.5)	0.697

SD indicates standard deviation.

At the 5-year follow-up, 7 study participants (3.6%) were found to have recurrent hernias. Additionally, 1 study participant who had a recurrent hernia 1-year postoperatively was lost to follow-up at 5 years. In order to calculate the verified, ever-recurrence in the study, this study participant was added to the data analysis for hernia recurrence. The ever-recurrence at 5 years was 4.7% (n = 8 after 171 hernia repairs, Table 2). The medical doctors (recurrence rate = 2.3%) was noninferior to the surgeons (recurrence rate = 7.3%) in terms of 5-year hernia recurrence. Of note, all but 1 recurrence detected at the 5-year follow-up presented asymptomatically. Additional information about the study participants with documented recurrent inguinal hernias is available in Table 3. At 5 years, there were more deaths in the surgeon group (24.7% vs 10.3% mortality rate in the medical doctor group), a statistically significant difference (*P* = 0.013). No deaths were known to be related to the hernia surgery or subsequent complications. The details of the study participants who died are outlined in Appendix 1, see http://links.lww.com/AOSO/A375.

**TABLE 2.** Hernia Recurrence and Mortality at 5 Years

Primary Outcome	Medical Doctor (n = 88)	Surgeon (n = 81)	Absolute Difference, % (95% CI)	*P*
Hernia recurrence, n (%)	2* (2.3)	6 (7.3)	−5.0 (−10.5)†	0.06
Death	9 (10.2)	20 (24.7)	−14.4 (−25.8 to −3.1)	0.013

* One hernia recurrence detected 1 year postoperatively and 1 hernia recurrence detected 5 years postoperatively. Denominator for hernia recurrence is 88.

† One-sided confidence interval for noninferiority hypothesis.

CI indicates confidence interval.

**TABLE 3.** Summary of Hernia Recurrences at 1 and 5 Years

	Age at Operation	Hernia Classification	Provider Type	1-Year Recurrence	5-Year Recurrence
1	73	Inguinoscrotal hernia, reduces with manual manipulation	Surgeon	No	Yes
2	45	Groin hernia, reduces with gentle pressure	Surgeon	No	Yes
3	46	Inguinoscrotal hernia, reduces with manual manipulation	Medical Doctor	Yes	Lost to follow-up
4	30	Groin hernia, reduces with gentle pressure	Surgeon	Yes	Yes
5	37	Inguinoscrotal hernia, reduces with manual manipulation	Surgeon	Yes	Yes
6	39	Groin hernia, reduces with gentle pressure	Medical doctor	No	Yes
7	56	Inguinoscrotal hernia, reduces with manual manipulation	Surgeon	Yes	Yes
8	64	Groin hernia, reduces spontaneously	Surgeon	No	Yes

There were no significant differences in the secondary outcomes of pain and self-assessed health status between the surgeon and medical doctor groups 5 years postoperatively (Table 4). Overall, 98.2% of participants (n = 166), were satisfied with the outcome of the surgery and all but 1 participant had less symptoms in the operated groin compared to before the operation. Mean IPQ scores for the medical doctor and surgeon groups were 1.32 and 1.29, respectively (*P* = 0.816). The distribution of IPQ scores was also similar between the 2 study groups, with 4.6% and 4.9% in the medical doctor and surgeon groups, respectively, experiencing pain that interferes with daily activities at 5 years postoperatively (*P* = 1). There were no wound complications or mesh infections observed in the participants presenting for 5-year follow-up.

**TABLE 4.** Secondary Outcomes at 5 Years

Outcome	No. (%)			
	Medical Doctor (n = 87)	Surgeon (n = 82)	Absolute Difference, % (95% CI)	*P*
Lesser degree of groin symptoms compared to before the operation	87 (100)	81 (98.8)	1.2 (−1.16 to 3.6)	0.302
IPQ score, mean (SD)	1.32 (0.90)	1.29 (0.76)	0.03 (−0.22 to 0.28)	0.816
Distribution of IPQ				
IPQ 1	73 (83.9)	69 (84.2)	−0.2(−11.3 to 10.8)	1
IPQ 2–3	10 (11.5)	9 (11.0)	0.5 (−9.0 to 10.0)	
IPQ 4–6	4 (4.6)	4 (4.9)	−0.3 (−6.7 to 6.1)	
Self-assessed health status score, mean (SD)	79.9 (15.9)	78.1 (16.9)	1.8 (−3.2 to 6.7)	0.501
Change from preoperative health status score, mean (SD)	10.5 (18.3)	10.2 (21.2)	0.3 (−5.7 to 6.3)	0.922
Patient satisfied with result of surgery	85 (97.7)	81 (98.8)	−1.1 (−5.0 to 2.9)	0.595

CI indicates confidence interval; SD, standard deviation.

**FIGURE 2.**
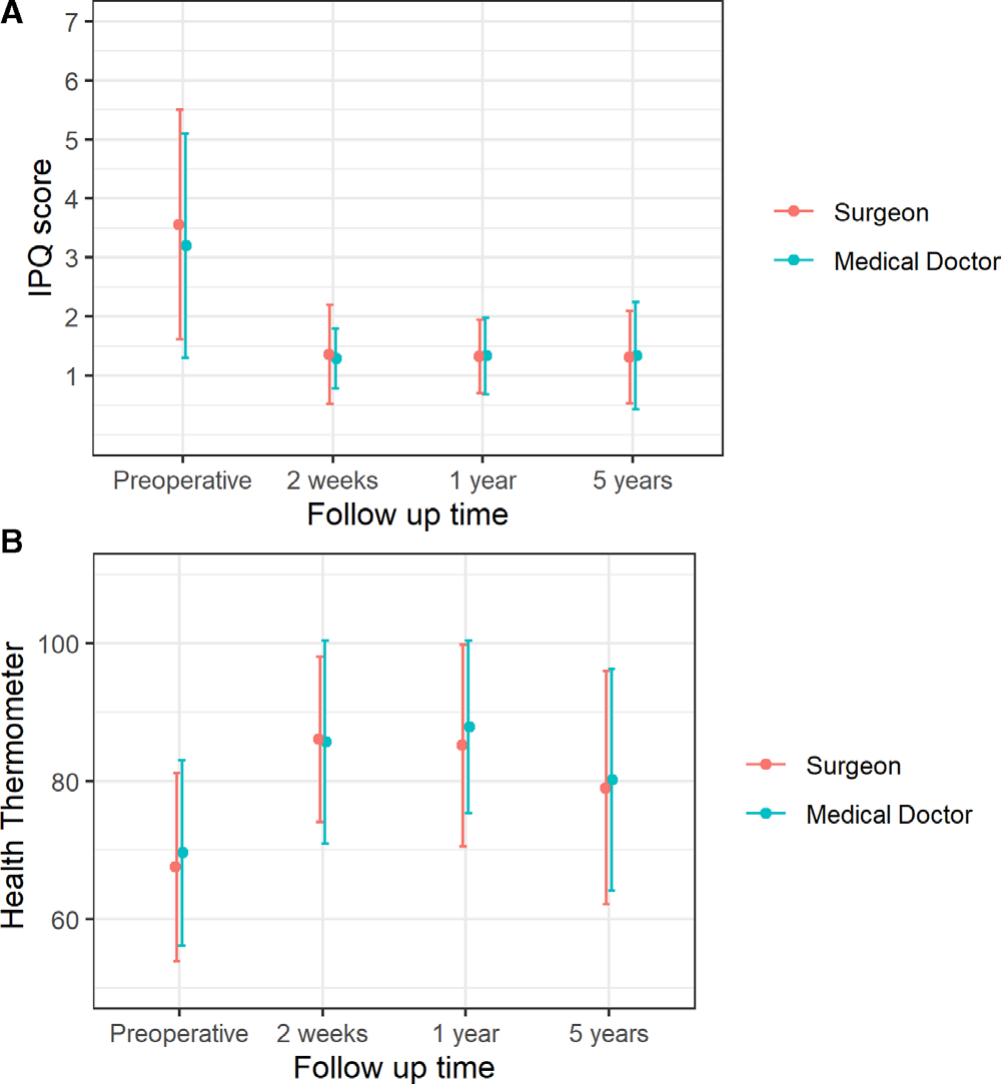
A, Inguinal Pain Score over time for patients operated on by surgeons and medical doctors. Level of pain according to IPQ reduced considerably directly after surgery and remained constant up to 5 years postoperatively. Only study participants with complete data were included in the data analysis. B, General health assessed by health thermometer (0–100) over time for patients operated on by surgeons and medical doctors. The general health of the study participants increased considerably directly after surgery, remained high at 1 year postoperatively but had reduced to some extent 5 years postoperatively. Only study participants with complete data were included in the data analysis.

The mean IPQ scores as well as the general health assessed by the health thermometer over time, including measurements preoperatively and at 2 weeks, 1 year, and 5 years postoperatively are shown in Fig. 2A, B, respectively. In these figures, only participants with complete data were included. Reduction of groin symptoms was seen 2 weeks postoperatively and persisted over time. Similarly, an immediate improvement in the self-assessed health status of participants was also observed; however, this health status gain did decrease somewhat over time.

## DISCUSSION

This cohort study represents one of the largest and longest-term follow-ups available examining outcomes of Lichtenstein inguinal hernia repair. Our results indicate that with adequate training and competency assessment, surgical providers in low-resource settings can achieve long-term recurrence rates following elective primary inguinal hernia repair with mesh similar to those observed in high-income countries. Our 5-year recurrence rate of 4.7 % compares favorably to long-term recurrence rates following Lichtenstein repair documented in studies carried out in Canada (2.0% at 5 years), Denmark (2.2%–3.8% at 3 and 6 years, respectively), Finland (2.5% at 7 years), United States (3.7% at 5 years), Netherlands (6.3% at 7 years), and Switzerland (8.1% at 6 years).^3,31–36^ Of note, because several of these comparator studies in high-income countries use hernia reoperation as a proxy for recurrence, their results likely underestimate the true rate of hernia recurrence.^3,31,32,34^ In addition, more than half of the participants in our cohort had inguinoscrotal hernias, which are more complex to repair and less common in high-income settings. Studies on outcomes following Lichtenstein hernia repair in LMICs are extremely limited. We are aware of only one study in sub-Saharan Africa examining long-term outcomes of mesh inguinal hernia repair. In that study, the 3-year recurrence rate following Lichtenstein repairs performed by a single surgeon in Northern Ghana was 6.8%, which also compares favorably with our results.^21^

The findings of this study provide further evidence of the safety and efficacy of task sharing of inguinal hernia repair with mesh between surgeons and medical doctors. While our previous work has found that medical doctors, associate clinicians, and surgeons can all be trained to perform high-quality hernia repair with mesh in low-resource settings, this study strengthens the evidence for this assertion with long-term outcomes.^19,20^ Going forward, we recommend that surgical capacity-building programs utilize task sharing to increase the number of clinicians available to provide high-quality hernia repair with mesh. It is important to note that only medical doctors and surgeons who were already performing hernia repairs were included in this study. The training of novice learners will require a more comprehensive educational intervention, which will be addressed in future research projects. Health policy planning activities should consider how best to utilize task sharing as a workforce development tool, and future studies should examine the safety and efficacy of task sharing for other essential surgical procedures.

Our study results add to the global literature on long-term outcomes following inguinal hernia repair with mesh. A significant reduction in groin pain was seen at 2 weeks following hernia surgery, and the improvement in IPQ score was sustained at 5 years postoperatively. This finding provides further evidence of the long-term effectiveness of Lichtenstein mesh repair in improving symptoms from inguinal hernia. In terms of severe chronic pain, the overall rate was 4.7%, which compares favorably to the incidence of chronic pain following Lichtenstein repair reported in high-income settings.^37^ Interestingly, the 8 participants who reported severe pain at the 5-year follow-up did not have recurrent hernias and all but 2 participants reported no pain at the 1-year follow-up. This suggests either that chronic pain following hernia repair can develop at a longer time interval or that the pain measured may be unrelated to the hernia surgery. In any case, the risk of severe chronic pain following Lichtenstein repair was not negligible.^37^ Therefore, providers must include this risk during informed consent discussions. Similarly, we found that the self-assessed health status of participants improved at 2 weeks following hernia repair. At the 5-year follow-up, the self-assessed health status score remained higher than preoperative measures, which is especially notable as participants likely developed other health concerns during that period. These findings highlight the value of measuring patient-centered outcomes following hernia surgery in addition to hernia recurrence and provide further evidence of long-term benefits of mesh inguinal hernia repair.

In this study, 18% of the study sample was lost to follow-up, mainly due to changes in participant contact information over the study period. At the 5-year follow-up, we used a variety of methods to locate the study participants, including calling them and their next of kin, contacting village elders, and in some cases traveling to their area of residence at the time of the surgery. For comparison, at the 1-year follow-up, only 5% of the study participants were lost to follow-up. Given this, it is possible that we could have reduced the rate of loss to follow-up at 5 years by regular communication with the study participants. In this study, ongoing communication was impeded by the global COVID-19 pandemic; however, regular contact with study participants could be considered in future research. Despite limitations and considerable challenges in locating the participants, our findings indicate that clinical studies that include long-term follow-up are possible in low-resource settings.

The 5-year mortality rate of 12% was higher than anticipated in this relatively healthy cohort. Notably, the ASA classification in this study was based on self-reported medical history and basic physical examination. In a setting where access to healthcare services is limited, many health conditions likely go unrecognized and untreated, suggesting that ASA classification by patient report and physical examination may overestimate a patient’s health status. In the review of the mortalities, we did not find any deaths related to the hernia or the hernia surgery; however, participants who died were 10 years older than those who attended the 5-year follow-up (61 vs 51 years, respectively). They were also more frequently smokers. For reference, the life expectancy for men in Ghana at birth is 62 years while life expectancy at 51 and 61 years is 25 and 17 years, respectively.^38,39^ It is likely that limited access to medical care during the COVID-19 pandemic along with undiagnosed medical conditions in our older participant cohort contributed to this relatively high rate of mortality observed in our study. In a resource-limited context, the cost-effectiveness of any medical intervention is important to consider. By definition, an inguinal hernia repair is more cost-effective in a younger compared to an older patient.^40^ Many of the study participants had lived with their hernias for years. To maximize the cost-effectiveness of inguinal hernia repair in Ghana and other low-resource settings, it is essential to improve access to hernia surgery, so that hernias can be repaired when they are identified and patients can benefit from this demonstrated health gain for as long as possible. Heath economics modeling has indicated that an additional 1200 surgical providers would be needed in order to eliminate the backlog of inguinal hernia in men in Ghana.^40^ Using task sharing between surgeons and medical doctors in Ghana, this ambitious goal could be achieved by 2030 at a total cost of USD 194 million, resulting in the avoidance of a significant burden of ill-health and premature deaths (1.5 million disability-adjusted life years averted).^40^

This study has several limitations. We did not randomize participants to treatment groups due to considerations by one of the institutional review boards. Instead, the study was designed as a prospective cohort, with patients assigned to the operating surgeon or medical doctor based on clinician availability within the structure of 4 high-volume surgical camps. Although there were no notable differences in baseline participant characteristics found in the 2 provider groups in the initial cohort analysis, it is possible that this design did introduce some unmeasured bias based on our 5-year outcomes.^19^ For example, the mortality rate among participants operated on by surgeons was significantly higher at 5 years than among participants operated on by medical doctors, suggesting the possibility of an unmeasured difference in the health status of participants in each of the provider cohorts. Similarly, the recurrence rate among participants operated on by medical doctors was lower than the recurrence rate found in surgeon patients at 5 years. Because it is unlikely that surgeons performed lower-quality hernia repairs than medical doctors, it is possible that unmeasured differences between these 2 groups owing to the nonrandomized design could explain these findings. This limitation does not mean that our results are invalid or negate the interpretation that task sharing is safe and effective. Task sharing implies that tasks are shared between medical doctors and surgeons and that more experienced providers offer oversight when needed to less experienced providers. The surgeries in this study were carried out in a real-world setting, which included opportunities for supervision for providers in both groups. As with our previous study, our results do not show that any medical doctor should be trained to perform mesh hernia repair in any hospital. Instead, our findings indicate that both medical doctors and surgeons with experience and aptitude for hernia surgery can be taught to perform mesh inguinal hernia repair safely and effectively with excellent long-term outcomes. The outcomes of this study indicate that the mortality rate, even in a generally healthy cohort of participants, is high in this setting. This mortality rate decreased the statistical power of this study, suggesting that future studies should include context-appropriate consideration of mortality rates in sample size calculations. Finally, our study included only adult men with reducible inguinal hernias; therefore, our results should not be generalized to include emergency hernia surgery or hernia repairs for women and children.

Surgeons and medical doctors trained as part of this study continue to provide mesh hernia repairs at the study site along with several other hospitals around Ghana. Since the initial program reported here, the Ghana Hernia Society has held similar courses aimed at training medical doctors working at first-level hospitals in mesh inguinal hernia repair. An evaluation of the impact of the Ghana Hernia Society mesh repair training program is ongoing. Additionally, the team is planning for repair for the patient who presented with a symptomatic recurrent hernia at 5 years.

## CONCLUSIONS

Task sharing of elective inguinal hernia repair with mesh for adult men with medical doctors is safe and effective in Ghana. Long-term outcomes of mesh hernia repairs performed by medical doctors in Ghana were excellent, with low rates of recurrence and significant improvements in pain and general health status following repair at 5 years postoperatively. We conclude that task sharing, with appropriate oversight, represents a critical tool to address the substantial morbidity related to unmet need for inguinal hernia surgery in Ghana. Going forward, commercial mesh needs to be made affordable and available for all patients with inguinal hernia, and further research focusing on task-sharing outcomes following surgery for groin hernia in women and children is warranted.

## ACKNOWLEDGMENTS

We acknowledge the administration, staff, and surgical team at Volta Regional Hospital for their assistance with study implementation and data collection. Ivette Raices Cruz, PhD (Karolinska Institutet) assisted with the statistical analysis and was compensated for her time.

## Supplementary Material


