# Supplementary material for: Long-Term Outcomes Following Inguinal Hernia Repair With Mesh Performed by Medical Doctors and Surgeons in Ghana
Source: Ann Surg Open. 2024 Jul 15;5(3):e460. doi: 10.1097/AS9.0000000000000460 (PMC11415131; doi:10.1097/AS9.0000000000000460)
Supplement: Supplementary file 1 [file as9-5-e460-s001.pdf]

Appendix 1. Summary of Mortalities at 5 Years

Age at Death	Reported Medical Problems	Time from Surgery to Death (years)	Likely Cause of Death
74	None	3	COVID-19
58	None	5	Trauma from fall
62	Hypertension	1	Unknown, complained of abdominal pain
59	None	5	Tuberculosis
78	None	3	Deep vein thrombosis/pulmonary embolus
69	None	4	Electrocution
66	None	1	Cerebrovascular Accident
62	None	4	Alcoholic liver disease
49	Hypertension	1	Heart failure
74	Hypertension	2	Severe anemia, Prostate cancer
61	None	1	Alcoholism, Dehydration
54	None	1	Unknown, found dead in bed
81	Hypertension	5	Cerebrovascular Accident
61	None	0.5	Subarachnoid hemorrhage
37	None	3	Unknown, difficulty breathing
71	None	5	Perforated peptic ulcer
71	None	0.5	Deep vein thrombosis/pulmonary embolus
43	None	5	Myocardial infarction
81	None	5	Unknown
78	None	2	CVA, Pneumonia
65	None	3	Renal Failure

55	None	5	Appendicitis, Heart Failure
54	None	3	Tuberculosis
72	None	5	Liver abscess
59	None	4	Unknown, abdominal pain and unable to walk
74	None	4	HIV/AIDS, weight loss
74	Hypertension	5	Urosepsis, dehydration
74	None	2	Fever, cough, chest pain
53	None	5	Hypertension
